# Tiger nut: Antidote for alcohol-induced testicular toxicity in male Sprague-Dawley rats

**DOI:** 10.5935/1518-0557.20210061

**Published:** 2022

**Authors:** Stella Chinwe Gbotolorun, Abiola Adebola Salako, Babatunde Ogunlade

**Affiliations:** 1 Anatomy Department, College of Medicine, University of Lagos, Lagos State, Nigeria; 2 Human Anatomy Department, Federal University of Technology Akure, Ondo State, Nigeria

**Keywords:** alcohol, tiger nuts, testicular toxicity, semen parameters, seminal contents of vitamins C and E, oxidative stress

## Abstract

**Objective:**

Studies have implicated alcohol consumption as a factor leading to male infertility. Tiger nuts (*Cyperus esculentus*) on the other hand, have been shown to possess the potential to boost male reproductive indices. This study was carried out to investigate the effect of tiger nuts on alcohol-induced testicular toxicity in male Sprague-Dawley rats.

**Methods:**

Thirty male Sprague-Dawley rats (160g averagely) were divided into six groups (A-F) (n=5). Group A (Control) received distilled water only; Group B (Tiger nut alone) received 1.8 g/kg body weight (BW) tiger nut; Groups C-F received 1 ml of 30% v/v alcohol three times weekly but groups C-E, also, received varied doses of tiger nut (0.6, 1.2 and 1.8 g/kg) (low, medium and high doses, respectively). All administrations were by oral gavage for 8 weeks. Serum was obtained and assayed for FSH, LH, and testosterone. Sperm was analyzed for semen parameters, and vitamins C and E contents. The testes were analyzed for antioxidants and histomorphology.

**Results:**

There was a significant decrease in body and testicular weights, semen characteristics with altered contents of vitamins C and E, hormone profiles, and testicular morphology in alcohol-exposed rats when compared with the control animals. However, the administration of tiger nuts improved the testicular architecture, semen parameters, and antioxidant enzymes in a dose-dependent manner.

**Conclusions:**

Supplementation with tiger nuts following alcohol administration produces a reversal of the deleterious effect of alcohol on the testis in a dose-dependent manner.

## INTRODUCTION

Alcohol is a well-known testicular toxicant causing part of its damage through increased lipid peroxidation (Duru *et al.,* 2006). Reports have demonstrated that excessive ethanol consumption can produce oxidative stress and induce testicular damage (Maneesh *et al.,* 2005). Moreover, an increased intake of ethanol is known to increase the levels of lipids, leading to hyperlipidemia (Balasubramaniyan *et al.,* 2003). Although significant progress has been made in understanding the pathogenesis of alcoholic testicular damage, treatment options are limited as well as problematic. Alcohol also reduces the synthesis of luteinizing hormone by its effect on the pituitary-gonadal axis (Bansal & Bilaspuri, 2010). Studies conducted on experimental animals also showed that a diet enriched with ethanol results in abnormal sperm parameters, morphological alterations involving the reproductive tract, and reduced mouse oocyte *in vitro* fertilization rate (La Vignera *et al.,* 2013). In humans, high alcohol intake is associated with serious disorders of spermatogenesis, such as lowered fertility, impaired testosterone production, testicular atrophy, erectile dysfunction, reduced male potency, and secondary sexual characteristics. Numerous studies have demonstrated that chronic alcohol consumption may cause reduced production of testosterone and lead to testicular shrinkage (i.e. testicular atrophy) in males (Adler, 1992). Those changes can result in impotence, infertility, and reduced male secondary sexual characteristics (e.g., reduced facial and chest hair, breast enlargement, and a shift in fat deposition from the abdomen to the hip area) (Anderson *et al*., 1983).

In recent times, researchers have developed an interest in the use of medicinal plants with antioxidant activity for protection against organ toxicities (El-Nekeety *et al*., 2009; Sudjarwo *et al*., 2017). Tiger nut, an underutilized crop of the family *Cyperaceae*, produces rhizomes and tubers that are somewhat spherical (Bamishaiye & Bamishaiye, 2011). It is an annual or perennial plant, growing to 90 cm (3.0 ft) tall. It can be eaten raw, dried, roasted, grated, or used for the production of nougat, jam, beer, or 'kunnu', a local non-alcoholic beverage in Nigeria (Belewu & Abodunrin*,* 2006). It is very rich in carbohydrates, mono- and di-fiber, and oil - especially oleic acid. Its moderately high level of protein, minerals, calcium, magnesium, iron and phosphorous, vitamins C and E makes it a good source of food for humans and animals (Arafat *et al.,* 2009). *Cyperus esculentus* (*C. esculentus*) has been used in the relief of diarrhea, dysentery, and indigestion (Abano & Amoah, 2011) since it contains numerous antioxidants, including vitamin E, Vitamin C, and Quercetin as well as minerals such as Zinc, potassium, and phosphorus (Allouh *et al*., 2015). *C. esculentus* is known to boost sexual competence and also increase male fertility by increasing libido and sexual performance, as well as restore sexual abnormalities (Allouh *et al*., 2015; Sabiu *et al*., 2016). Improvements in sperm count, motility, viability, and morphology, testicular architectural maintenance, and increased testosterone concentration has been reported following administration of *C. esculentus* (Ekaluo *et al*., 2015; Atoigwe-Ogeyemhe *et al*., 2018). Tiger nut methanolic extract has been reported to improve sperm count and motility in male rats, and it is associated with increased gonadotropins and testosterone levels (Al-Shaikh *et al.,* 2013; Agbai & Nwanegwo, 2013). However, very little or nothing is known about the effect of tiger nuts co-administered with alcohol on the testis. Therefore, this study was carried out to investigate the effects of tiger nuts as daily supplements on alcohol-induced testicular injury in male Sprague-Dawley rats.

## MATERIALS AND METHOD

### Alcohol

Alcohol (99.9%) was procured from a Pharmacy store in Mushin, Lagos, Nigeria. We prepared 30% v/v of alcohol by diluting 30 ml of alcohol in 70 ml of distilled water. We administered the prepared solution orally at a dose of 1 ml three times a week (Mondays, Wednesdays, and Fridays) for eight weeks.

### Tiger Nut Extract

Fresh tiger nuts were purchased from the local market in Yaba, Lagos, Nigeria. The tiger nuts were identified and authenticated by Professor J.D. Olowokudejo of the Department of Botany, University of Lagos, Nigeria, and were deposited in the herbarium with Voucher specimen No-LUH8060. The tiger nuts were washed thoroughly to remove sand particles and dirt, then air dried, and pulverized into powder using a blender. Tiger nut suspension was made by adding distilled water. Three different doses of tiger nut suspension (0.6, 1.2, and 1.8 mg/kg body weight) were administered immediately after alcohol administration.

### Experimental animals

Thirty-five adult male Sprague-Dawley rats with an average weight of 160 g were procured from the Animal House - College of Medicine of the University of Lagos, Nigeria. They were housed in the animal house of the department of Anatomy in well-ventilated plastic cages. They were left to acclimatize for two weeks to get accustomed to the new environment. The rats were fed with standard rat chow and water *ad-libitum*. Body-weight changes of the animals were measured weekly throughout the study. All procedures were carried out following the National Academy of Science Guide for care and use of laboratory animals (National Research Council, 1996), and were approved by the Health Research Ethics Committee of the College of Medicine, University of Lagos, Nigeria sought under protocol number CMUL/HREC/02/19/500.

### Experimental design

The animals were randomly divided into six groups of five animals each, as described below:

1. Group A (CONTROL) received 1 ml distilled water
2. Group B (TIGER NUT) received 1.8 mg/kg body weight tiger nut only
3. Group C (ALC + LDTN) received 1 ml alcohol + 0.6 mg/kg body weight tiger nut
4. Group D (ALC + MDTN ) received 1 ml alcohol + 1.2 mg/kg body weight tiger nut
5. Group E (ALC + HDTN) received 1 ml alcohol + 1.8 mg/kg body weight tiger nut
6. Group F (ALC) received 1 ml alcohol only.

Alcohol was administered three times a week for 8 weeks (time to complete a spermatogenic cycle). A fresh suspension of tiger nut was made daily and all administrations were given orally by gavage. The choice of the administered doses of tiger nut was determined based on previously published work (Ekaluo *et al.,* 2015).

### Animal sacrifice

At the end of the study, the animals were slaughtered by cervical dislocation. Blood samples were taken by cardiac puncture into plain bottles for hormonal assay. The serum was separated by allowing the blood sample to stand for 15 minutes at 25ºC and then centrifuged at 4000 rpm for 20 minutes. The serum was kept in plastic vials and stored in a refrigerator at -40ºC until further analysis. The Testes were excised, rinsed in cold saline, blotted dry, and weighed. The epididymis was excised and seminal fluid was analyzed for semen parameters, vitamins C and E. Thereafter, the right testes were immediately fixed in bouin's fluid for histology, while the left testes were stored in the refrigerator at -20ºC for analysis of antioxidant status.

### Tissue processing for histology

The fixed testicular tissues were processed for microscopic examination using a standard protocol, and 5 µm thick paraffin sections were made using the Reichert Ultra Microtome. We stained the slides with routine Hematoxylin and Eosin, according to the routine procedure for light microscopy, and we took the photomicrographs using Olympus and Leica microscopes

### Testicular homogenate

The testicular tissue was homogenized with a mortar and pestle, in 2.5 ml of 0.15M KCl. The homogenate was centrifuged at 1000 rpm for ten minutes and we collected the supernatant. We then added a 2ml aliquot of thiobarbituric acid (0.375% 1 mol/L), 15% trichloroacetic acid to a 1 ml of the tissue supernatant, mixed vigorously, and heated for 15 minutes in boiling water (80-90ºC). The samples were cooled in ice-cold water and centrifuged at 1500 rpm for 15 minutes.

### Oxidative stress markers analyses

Superoxide dismutase (SOD) was assayed by the method described by Sun *et al.* (1988).

Lipid peroxidation was quantified as malondialdehyde (MDA), according to the method described by Placer *et al.* (1966), and it was expressed as micro-molar of MDA per gram of tissue. Catalase (CAT) was assayed calorimetrically at 620 nm and expressed as µmoles of H_2_O_2_ consumed/min/mg/protein, as described by Rukkumani *et al.* (2004). The reduced glutathione (GSH) level in testicular tissue was estimated as described by Rukkumani *et al.* (2004).

### Hormonal assays

The serum levels of Follicle-stimulating hormone (FSH), Luteinizing hormone (LH), and Testosterone were determined based on the principle of rat Enzyme-linked Immuno-Assay (ELISA), as described by the LSBio^®^ procedure, with kit catalog numbers LS-F6305, MBS72998730S, and EIA- 3069, respectively.

### Semen parameter determination

The caudal epididymis of each animal was excised and placed in a small clean Petri dish containing 1 ml of phosphate buffer saline at a pH of 7.4, and the concentration was then allowed to settle at 37ºC for 10 minutes (Anjamrooz *et al.,* 2007). The sperm count and progressive motility were determined four times for each sample (Cheng *et al.,* 2007) using the improved Neubaur-hemocytometer Chamber (Deep 1/10 mm, LABART, Germany), as described by Pant & Srivastava (2003). We carried out microscopic examinations of the seminal smears stained with Eosin Nigrosine stain to determine sperm morphology. We used a binocular microscope (Leica DM 750, Switzerland) at 40× and 100× magnifications.

### Vitamins C and E determination in the seminal plasma and spermatozoa

We used high-performance liquid chromatography (HPLC) to measure the concentrations of vitamin C and E in the seminal plasma and spermatozoa.

### Vitamin E measurements

We measure vitamin E according to the method by Lee *et al.* (Lee *et al.,* 1992). Briefly, an aliquot of the seminal plasma was placed into a sample preparation vial, which was filled with Sodium Sulfate for precipitation. We then added a stabilizing reagent, butanol ethyl acetate (1:1). We removed the solid component of the seminal plasma precipitated by centrifugation. Finally, 20 µl of the supernatant was injected into the HPLC system. Detection was done by fluorescence at 286 nm.

### Vitamin C measurement

Seminal and spermatozoa ascorbic acid was determined using conventional methodology. Briefly, ascorbic acid in the sample was converted to dehydroascorbate in the presence of thiourea and copper sulfate. We mixed 0.5 ml samples with 0.4 ml DTCs (containing thiourea, copper sulfate, and DNPT) and incubated for 3 hours at 37ºC. Dehydroascorbate was then coupled with 2,4-dinitrophenyl hydrazine forming its bis derivative. Upon treatment with 2 ml sulphuric acid (12 mol), the derivative yields a stable red color, which was measured spectrophotometrically at 520 nm (Natelson, 1971).

### Statistical Analysis

The results were analyzed using the statistical package for the social sciences version 21.0 (SPSS, Chicago, IL, USA). We ran the statistical analysis using the paired sample test and one-way Analysis of Variance (ANOVA). The data were reported as mean ± Standard Error of Mean (S.E.M) and multiple comparisons were done using the LSD post - hoc tests. The significant difference was determined at 0.05 levels (*p*<0.05).

## RESULTS

### Effect of Alcohol and supplementation with Tiger nut on seminal parameters and testicular weight in male Sprague-Dawley rats

The result showed a significant (*p*<0.05) decrease in sperm motility in the ALC + LDTN, ALC + MDTN, and ALC alone groups when compared to the TN alone and control groups. Of these 3 groups, the ALC alone group showed further significant reduction compared to the other 2 groups. Sperm count significantly (*p*<0.05) reduced when the ALC alone treated group was compared to all the treatment groups; but when the ALC + TN treatment groups were compared to the TN alone group, sperm count reduced significantly in the low and medium dose groups. Sperm abnormal morphology increased significantly when the ALC alone treated group was compared to all other treatment groups (Table 1). Testicular weight significantly increased in the TN alone treated group compared with the control; while testicular weights decreased significantly when the ALC + LDTN, ALC + MDTN, and ALC alone groups were compared to the TN alone group (Table 1).

**Table 1.** Effect of alcohol and tiger nut on testicular weight and seminal parameters in adult male Sprague-Dawley rats.

Groups	Sperm Motility (%)	Sperm Count (10^6^/ml)	Abnormal Morphology (%)	Testicular Weight (g)
CONTROL	92.4±2.25	76±3.02	0.6±0.24	1.20±0.12
TN	93.2±2.51	85.4±3.98	0.2±0.2	2.21±0.39*
ALC + LDTN	52±4.64^αβ^	69±3.18^β^	1.4±0.98	1.29±0.09^β^
ALC + MDTN	56±6.21^αβ^	63.6±2.32^β^	3±0.45^αβ^	1.21±0.02^β^
ALC + HDTN	80.6±4.73^γδ^	76.8±2.04^δ^	1.4±0.24	1.56±0.24
ALC	19±4.30αβγ^δε^	48±2.17αβγ^δε^	6.4±0.51^αβγδε^	1.07±0.06^β^

Values are expressed as mean ± Standard Error of Mean (S.E.M).

α *p*<0.05 significant when compared to control.

β *p*<0.05 significant when compared to TN group.

γ *p*<0.05 significant when compared to ALC + LDTN group.

δ *p*<0.005 significant when compared to ALC + MDTN group.

ε *p*<0.05 significant when compared to ALC + HDTN group.

TN= Tiger nut alone (1.8 mg/kg bodyweight of Tiger nut); ALC + LDTN= Alcohol + Low Dose Tiger nut (0.6 mg/kg body weight of Tiger nut); ALC + MDTN= Alcohol + medium dose Tiger nut (1.2 mg/kg body weight); ALC + HDTN= Alcohol + high dose Tiger nut (1.8 mg/kg bodyweight); ALC= Alcohol alone (1 ml Alcohol three times a week).

### Effect of alcohol and supplementation with tiger nut on the seminal fluid concentration of vitamins C and E in Sprague-Dawley rats

Seminal fluid concentrations of vitamin C and E showed similar results in the treatment groups. TN alone groups showed significantly (*p*<0.05) increased values when compared to the control group, while the ALC alone group, on the other hand, reduced significantly in comparison with all the other treatment groups. Furthermore, ALC + LDTN and ALC + MDTN groups showed significantly reduced concentrations of vitamins C and E when compared to the TN alone treated group (Table 2).

**Table 2.** Effect of alcohol and tiger nut on seminal fluid concentration of vitamins C and E in adult male Sprague-Dawley rat.

TREATMENT GROUPS	VITAMIN C (µmol/L)	VITAMIN E (µmol/L)
CONTROL	1.40±0.44	1.60±0.22
TN	2.90±0.08^α^	3.08±0.07^α^
ALC + LDTN	1.08±0.38^β^	0.80±0.41^β^
ALC + MDTN	1.11±0.28^β^	1.08±0.46^β^
ALC + HDTN	2.17±0.22	1.99±0.58
ALC	0.12±0.10^αβγδε^	0.03±0.01^αβγδε^

Values are expressed as mean ± Standard Error of Mean (S.E.M).

α *p*<0.05 significant when compared to control.

β *p*<0.05 significant when compared to TN.

γ *p*<0.05 significant when compared to ALC + LDTN.

δ *p*<0.005 significant when compared to ALC + MDTN.

ε *p*<0.05 significant when compared to ALC + HDTN.

TN= Tiger nut alone (1.8 mg/kg bodyweight of Tiger nut); ALC + LDTN= Alcohol + Low Dose Tiger nut (0.6 mg/kg bodyweight of Tiger nut); ALC + MDTN= Alcohol + medium dose Tiger nut (1.2 mg/kg bodyweight); ALC + HDTN=Alcohol + high dose Tiger nut (1.8 mg/kg body weight); ALC= Alcohol alone (1 ml Alcohol three times a week).

### Effect of alcohol and supplementation with tiger nut on the reproductive hormones of adult male Sprague-Dawley rats

The result showed significantly (*p*<0.05) decreased levels of FSH in the ALC + LDTN and ALC-alone treated groups when compared to the control animals. LH recorded significant decreases when the ALC-alone treated group was compared to the control, ALC + MDTN, and ALC + HDTN groups (Table 3). There was a significant increment in testosterone levels when the TN-alone and ALC + HDTN groups were compared with the control group. Furthermore, ALC + LDTN, ALC + MDTN, and ALC-alone treated groups recorded significant reductions in testosterone levels when compared with the TN alone treated group (Table 3).

**Table 3.** Effect of alcohol and tiger nut on the reproductive hormones of adult male Sprague-Dawley rats.

TREATMENT GROUPS	FSH (mIU/mL)	LH (IU/L)	Testosterone (ng/dL)
CONTROL	533.7±161.4	146.5±24.40	11.45±0.82
TN	317.1±74.23	115.8±29.19	31.21±9.49^α^
ALC + LDTN	132.1±17.04^α^	97.27±27.94	12.87±2.53^β^
ALC + MDTN	319.9±75.87	147.7±40.17	15.77±1.10^β^
ALC + HDTN	497.9±116.9	167.1±29.60	18.36±1.54^α^
ALC	97.84±10.73^α^	23.84±7.57 ^αδε^	10.88±0.54^βε^

Values are expressed as mean ± Standard Error of Mean (S.E.M).

α *p*<0.05 significant when compared to control.

β *p*<0.05 significant when compared to TN.

δ *p*<0.005 significant when compared to ALC + MDTN.

ε *p*<0.05 significant when compared to ALC + HDTN.

TN= Tiger nut alone (1.8 mg/kg body weight of Tiger nut); ALC + LDTN= Alcohol + Low Dose Tiger nut (0.6 mg/kg body weight of Tiger nut); ALC + MDTN= Alcohol + medium dose Tiger nut (1.2 mg/kg body weight); ALC + HDTN= Alcohol + high dose Tiger nut (1.8 mg/kg body weight); ALC= Alcohol alone (1 ml Alcohol three times a week).

### Effect of alcohol and supplementation with tiger nut on testicular oxidative stress markers in adult male Sprague-Dawley rats

Glutathione reductase (GSH) activity showed a significant (*p*<0.05) increase when TN alone, ALC + MDTN, and ALC + HDTN groups were compared to the control but showed a significant decrease when ALC + LDTN and ALC alone groups were compared to the control. MDA levels did not show any significant differences except in the ALC alone treated group, which decreased significantly when compared to the other treatment groups (Table 4). Catalase showed a significant increase when the TN alone group was compared to the control group, and significantly decreased when the ALC + LDTN and ALC alone groups were compared to the TN alone group. SOD showed a significant increase when the TN alone group was compared to the control animals, and a significant decrease when all the ALC + TN treatment groups were compared to TN alone group. Meanwhile, the ALC alone group showed significant reductions compared to both the control and TN alone groups (Table 4).

**Table 4.** Effect of alcohol and tiger nut on testicular oxidative stress markers in adult male Sprague-Dawley rat.

Groups	GSH (µ/mg)	MDA (µ/mg)	CAT (µ/mg)	SOD (µ/mg)
CONTROL	2.6±0.71	1.62±0.09	72.45±12.31	15.63±3.93
TN	6.14±0.6^α^	1.14±0.17	156.4±19.9^α^	65.77±8.46^α^
ALC + LDTN	1.71±0.15^β^	1.33±0.04	84.48±12.67^β^	15.36±1.84^β^
ALC + MDTN	4.65±0.67^αγδ^	1.16±0.21	90.51±14.17	25.03±3.99^β^
ALC + HDTN	4.65±0.67^γδ^	1.13±0.10^αβδ^	114.30±21.95	26.59±3.73^β^
ALC	0.96±0.17^β^	2.47±0.29^αβγδε^	53.15±9.38^β^	13.14±1.83^αβ^

Values are expressed as mean ± Standard Error of Mean (S.E.M).

α *p*<0.05 significant when compared to control.

β *p*<0.05 significant when compared to TN.

δ *p*<0.005 significant when compared to ALC + MDTN.

ε *p*<0.05 significant when compared to ALC + HDTN.

TN= Tiger nut alone (1.8 mg/kg body weight of Tiger nut); ALC + LDTN= Alcohol + Low Dose Tiger nut (0.6 mg/kg body weight of Tiger nut); ALC + MDTN= Alcohol + medium dose Tiger nut (1.2 mg/kg body weight); ALC + HDTN= Alcohol + high dose Tiger nut (1.8 mg/kg body weight); ALC= Alcohol alone (1 ml Alcohol three times a week).

### Effect of alcohol and supplementation of tiger nut on the histology of the testes of adult male Sprague-Dawley rats

**Figure 1:**
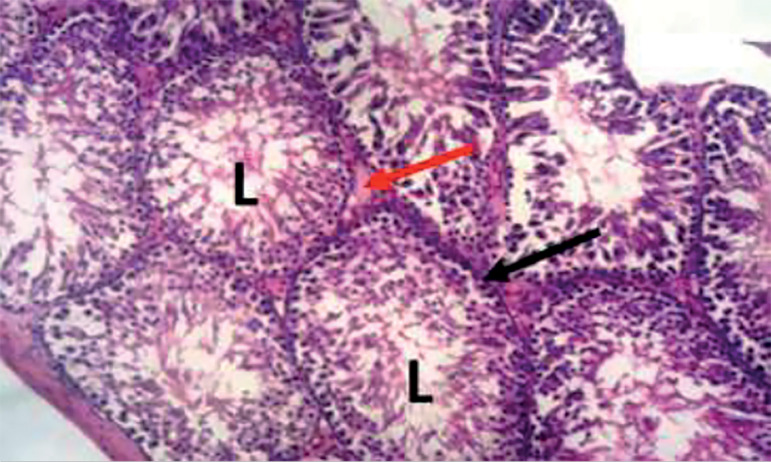
Photomicrograph of the testes of a control rat showing normal testes with typical seminiferous tubule, lumen (L), basement membrane (black arrow), and interstitial space (red arrow). H&E X 100.

**Figure 2:**
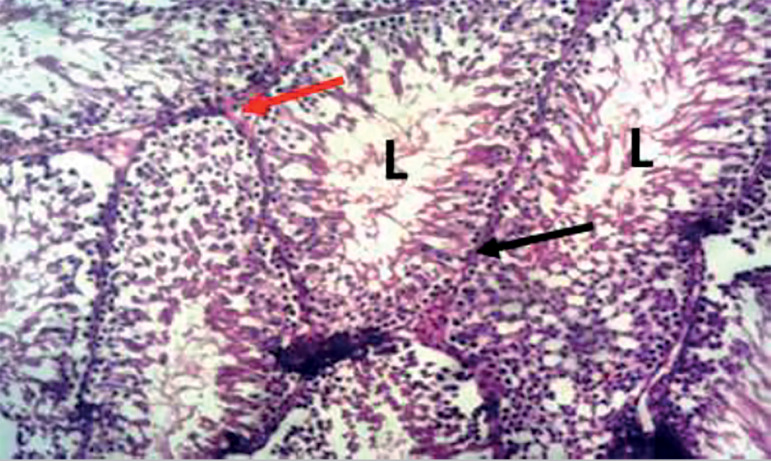
Photomicrograph of the testes from a tiger nut alone treated rat, showing typical seminiferous tubule, demonstrating the lumen (L) basement membrane (black arrow) and interstitial space (red arrow). H&E X100.

**Figure 3:**
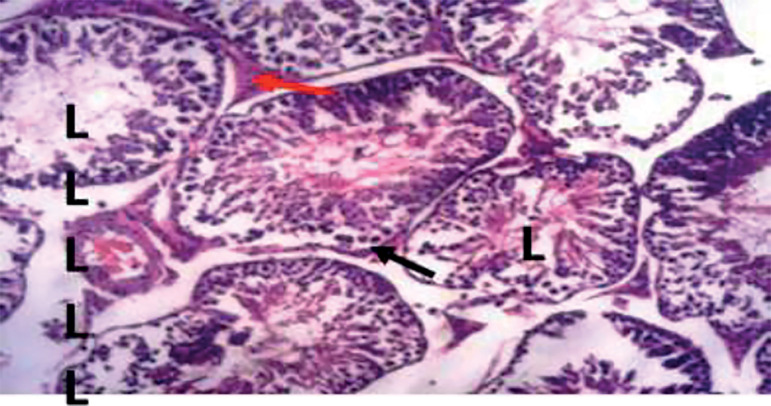
Photomicrograph of the testes of alcohol alone treated rat showing tubular degeneration with lacerated basement membrane (black arrow) presenting with histoarchitecture distortion. Lumen (I), is seen devoid of spermatids, interstitial space (red arrow) appear widened with destruction of interstitial cells. H&E X 100.

**Figure 4:**
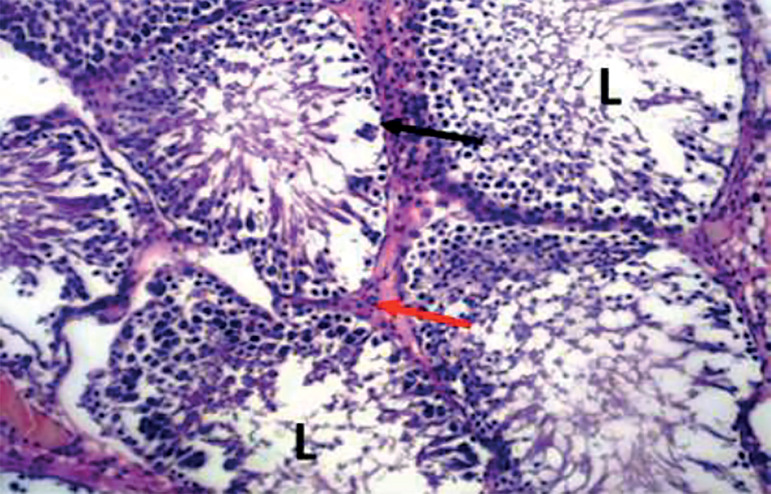
Photomicrograph of the testes of rat treated with alcohol + LDTN showing lacerated and degenerated seminiferous tubules, interstitial spaces (red arrow) appear somewhat widened compared to control. Lumen (L) appear devoid of spermatids. H&E X 100.

**Figure 5:**
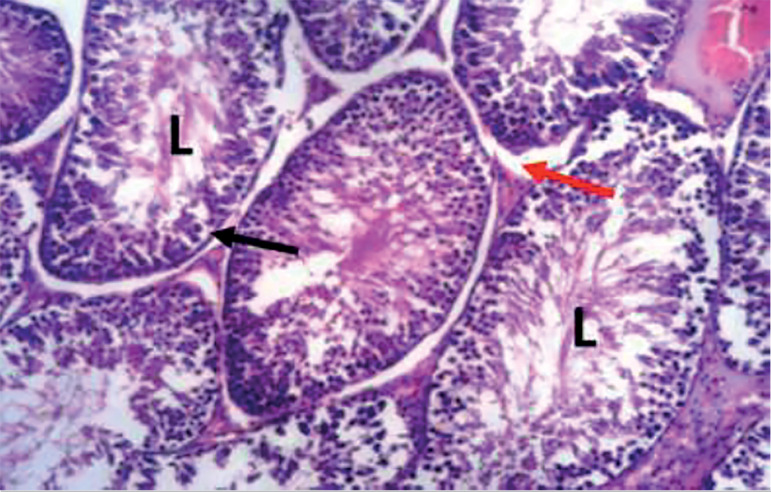
Photomicrograph of the testes of rat treated with ALC + MDTN showing mild tubular degeneration. The basement membrane (black arrow) and interstitial space (red arrow) appeared normal. H&E X 100.

**Figure 6:**
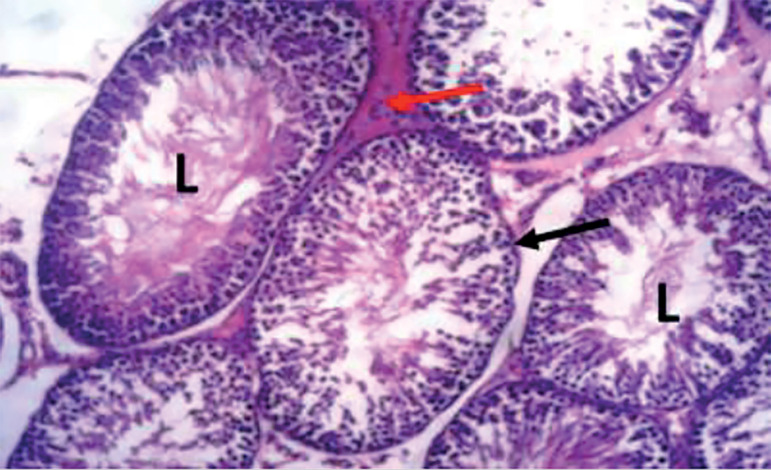
Photomicrograph of the testes of rat treated with ALC + HDTN showing normal seminiferous tubules, intact basement membrane (black arrow), normal interstitial spaces (red arrow) and lumen (l) filled with spermatids. H&E X 100.

The photomicrograph of cross-sections of the testes of the control group appeared normal and showed closely packed seminiferous tubules with a well-organized arrangement of the germinal epithelium. The interstitial spaces (IS) containing the Leydig cells appeared normal, basement membranes (BM) also appeared normal and intact. Large numbers of normally dividing spermatids were seen extending their cellular processes into the lumen (L) of the seminiferous tubules (Figure 1). Photomicrograph of the testes of the group treated with tiger nut alone revealed normal seminiferous tubules (Figure 2), similar in architecture to the control (Figure 1). When the alcohol-alone treated group was compared to the control and tiger nut alone groups, there was tubular degeneration, germinal epithelial disintegration, lacerated basement membranes (BM), widened interstitial spaces (IS) with the destruction of interstitial cells. In addition, the lumen (L) contained little or no spermatids (Figure 3). Sections from the ALC + TN treatment groups showed a reversal of the deleterious effect of alcohol on the testes in a dose-dependent manner. Sections from the ALC + LDTN group showed seminiferous tubules that appeared lacerated and degenerated, somewhat similar to the alcohol alone group, except with better-preserved interstitial spaces and interstitial cells (Figure 4). Aside from germinal epithelial disintegration, found in sections from rats treated with ALC + MDTN (Figure 5), the tubules appeared better preserved than the ALC + LDTN group. The histoarchitecture of the ALC + HDTN tubules appeared similar (Figure 6) to the control group.

## DISCUSSION

Several medicinal plants with antioxidant potential help in the preservation of various organs against drug toxicities (El-Nekeety *et al*., 2009; Sudjarwo *et al*., 2017). *Cyperus esculentus* (*C. esculentus*) (Tiger nut) is one of such plants with numerous antioxidants including vitamin E, Vitamin C, and Quercetin as well as minerals such as zinc, potassium, and phosphorus (Allouh *et al*., 2015). Numerous studies have demonstrated that chronic alcohol consumption can induce reduced production of testosterone and lead to testicular shrinkage (i.e. testicular atrophy) in males (Adler, 1992). Such changes can result in impotence, infertility, and reduced male secondary sexual characteristics (e.g., reduced facial and chest hair, breast enlargement, and a shift in fat deposition - from the abdomen to the hip area) (Anderson *et al*., 1983).

Testicular weight loss results primarily from the loss of sperm cells and decreased diameter of the seminiferous tubules (Van Thiel *et al*., 1974). From this study, we can deduce that the rats treated with alcohol only showed a significant decrease in testicular weight when compared to the control animals and the rats treated with Tiger nut. Testicular weight loss also appears to be common among alcoholics, occurring in up to 75% of men with advanced alcoholic cirrhosis (Lloyd & Williams, 1948). On co-administration of alcohol and varying doses of Tiger nut, the testicular weight was significantly restored when compared with the alcohol-only treated group. Karawya & El-Nahas (2006) previously reported increased testicular weight following *C. esculentus* administration after lead-induced testicular weight reduction, thereby corroborating our findings.

Furthermore, the testicular concentration of the rats administered with alcohol was significantly lower when compared to the control and the Tiger nut treated groups. Studies have reported a reduction in sperm count and concentration in mice and a disruption in spermatogenesis in rats following ethanol administration. Elgawish & Abdelrazek (2014) previously reported that the treatment of rats with ethanol resulted in decreased sperm concentration and viability, and increased sperm abnormal morphology. On co-administration of alcohol and varying doses of Tiger nut, the testicular concentration was significantly increased when compared to the alcohol-only treated rats. Consistent with previous studies (Al Essawe & Almashhadani, 2010; Al-Shaikh *et al*., 2013), the rats administered with *C. esculentus* extract only had a significantly increased sperm count, motility, and viability, and decreased abnormal morphology of sperm in comparison with the normal control rats, indicating the ability of the extract to improve sperm function.

Previous studies have shown the significance of antioxidants (Vitamins A, E, D, and C) in the field of andrology through their inhibitory role on ROS accumulation among other functions (Makker *et al*., 2009; El-Tohamy & El-Nattat, 2010). Vitamin C (L-Ascorbic acid) derived from dietary sources such as fruits and vegetables are water-soluble, and play an efficient protective role directly or indirectly in the systemic detoxification of several drugs and environmental toxicants (Sugiyama, 1992; Adeniyi *et al*., 2008). Additionally, Vitamin E is a lipid-soluble, chain-breaking antioxidant that plays a significant role in the inhibition of oxidation and oxidative damage to membrane polyunsaturated fatty acid (Sugiyama, 1992; Ayinde *et al*., 2012). The contents of these aforementioned vitamins in this study revealed a significant decrease in the alcohol-only group compared with the treatment and other groups. The accumulation of lipid peroxidation attributed to alcohol intoxication caused the formation of free radicals after depletion of antioxidants, thereby altering the function of some antioxidant enzyme markers, which corroborate our studies (Salawu *et al*., 2009; Vigeh *et al*., 2011). However, the Tiger nut groups showed significant elevation in the seminal content of Vitamins C and E, thereby protecting sperm DNA from the oxidative stress of free radicals and improving fertility (Tarín *et al*., 1998; Ayinde *et al*., 2012).

Exposure of rats to toxicants can result in a marked decrease in the levels of testosterone, LH, and FSH. A decrease in the levels of these hormones is an indication of spermatogenesis impairment (Guyton & Hall, 2000). A decrease in testosterone production is evidence of male reproductive toxicity (Yoshida *et al*., 2002). LH, secreted by the anterior pituitary gland, induces testosterone secretion by the Leydig cells. Furthermore, the quantity of testosterone secreted increases approximately in direct proportion to the amount of LH available; therefore, LH acts through a feedback mechanism to maintain testosterone biosynthesis (Guyton & Hall, 2000). From this result, reproductive hormone (Testosterone, FSH and LH) levels were significantly decreased in alcohol-only treated rats when compared to the control and Tiger nut treated rats. However, on co-administration of alcohol and varying doses of Tiger nut, the reproductive hormone (Testosterone, FSH and LH) levels were significantly increased when compared to the alcohol-only treated rats. The ability of the extract to increase reproductive hormone levels could be due to the presence of antioxidants like quercetin, vitamins C and E, and trace element such as zinc in *C. esculentus* (Al Essawe & Almashhadani, 2010; Al-Shaikh *et al*., 2013). These phytochemicals have powerful antioxidant properties, and have been reported to be associated with improvements in serum testosterone levels in male rats and mice (Dissanayake *et al*., 2009; Mohammadirad *et al*., 2013).

Oxidative stress is one of the common mechanisms implicated in testicular toxicity. Oxidative stress results when the production of reactive oxygen species (ROS) exceeds the capacity of the antioxidant defense system (Patrick, 2006). From this study, we can deduce that there is a significant decrease in CAT, SOD, and GSH levels, and a significant increase in the MDA level in the rats administered with alcohol only when compared to the control and Tiger nut treated rats. However, on co-administration of alcohol and varying doses of Tiger nut, a significant increase in CAT, SOD, and GSH levels; and there was a significant decrease in the MDA level when compared to the alcohol-treated rats. The ability of the extract to attenuate the effects of oxidative stress could be due to the presence of antioxidants like quercetin, vitamins C and E, and trace elements such as zinc in *C. esculentus* (Al Essawe & Almashhadani, 2010; Al-Shaikh *et al*., 2013). These phytochemicals have powerful antioxidant properties and have been reported to be associated with improvements in serum testosterone levels in male rats and mice (Dissanayake *et al*., 2009; Mohammadirad *et al*., 2013).

The histological observation of the testes in this study showed an abnormal testicular histoarchitecture in rats treated with alcohol only when compared with the control and Tiger nut treated rats evident by the absence of spermatids, wide interstitial spaces, and lumen. However, on co-administration of alcohol and varying doses of Tiger nut, the testicular histoarchitecture was normal and evident by normal seminiferous tubules, intact basement membrane, normal interstitial spaces, and lumen filled with spermatids, indicating that the extract exhibited the ability to ameliorate the decreased spermatogenesis and impaired histoarchitecture caused by the alcohol administration. The effect of the extract on the testicular morphometric parameters was dose-dependent, as high doses showed a more potent result than medium and low doses, and could be attributed to its antioxidant content (Patrick, 2006).

## CONCLUSION

The results of this present study have shown that the aqueous extract of tiger nut may increase sperm motility and count, testicular antioxidant status, testicular vitamins C and E concentrations, and testosterone levels. This study has demonstrated that at higher doses, the aqueous extract of tiger nut could protect the testes against the deleterious effects of alcohol and bring about the regeneration of the alcohol-damaged testicular milieu.

## List of abbreviations

FSH: Follicle stimulating hormone
TT: Testosterone
LH: Luteinizing hormone
SOD: Sodium dismutase
CAT: Catalase
GSH: Reduced glutathione
MDA: Malondialdehyde
ALC: Alcohol
LDTN: Low dose tiger nut
MDTN: Medium dose tiger nut
HDTN: High dose tiger nut
BM: Basement membrane
